# Leprosy piRnome: exploring new possibilities for an old disease

**DOI:** 10.1038/s41598-020-69355-7

**Published:** 2020-07-28

**Authors:** Pablo Pinto, Moisés Batista da Silva, Fabiano Cordeiro Moreira, Raquel Carvalho Bouth, Angélica Rita Gobbo, Tatiana Vinasco Sandoval, André Mauricio Ribeiro-dos-Santos, Amanda Ferreira Vidal, Josafá Gonçalves Barreto, Sidney Santos, John Stewart Spencer, Claudio Guedes Salgado, Ândrea Ribeiro-dos-Santos

**Affiliations:** 1 Human and Medical Genetics Laboratory, Institute of Biological Sciences (ICB), UFPA, Belém, 66075110 Brazil; 2 Oncology Research Center (NPO), UFPA, Belém, 66075110 Brazil; 3 Dermato-Immunology Laboratory, Institute of Biological Sciences (ICB), UFPA, Marituba, 67200000 Brazil; 4 Laboratory of Space Epidemiology (LabEE), UFPA, Castanhal, 68746000 Brazil; 5 Mycobacteria Research Laboratories, Department of Microbiology, Immunology and Pathology, Colorado State University, Fort Collins, 80523-1601 USA

**Keywords:** Gene regulation, RNAi, Sequencing, Next-generation sequencing, RNA sequencing, Bacterial infection, Demyelinating diseases

## Abstract

Leprosy, which is caused by the human pathogen *Mycobacterium leprae*, causes nerve damage, deformity and disability in over 200,000 people every year. Because of the long doubling time of *M. leprae* (13 days) and the delayed onset of detectable symptoms, which is estimated to be approximately 3–7 years after infection, there is always a large percentage of subclinically infected individuals in the population who will eventually develop the disease, mainly in endemic countries. piRNAs comprise the largest group of small noncoding RNAs found in humans, and they are distinct from microRNAs (miRNAs) and small interfering RNAs (siRNAs). piRNAs function in transposon silencing, epigenetic regulation, and germline development. The functional role of piRNAs and their associated PIWI proteins have started to emerge in the development of human cancers and viral infections, but their relevance to bacterial diseases has not been investigated. The present study reports the piRNome of human skin, revealing that all but one of the piRNAs examined are downregulated in leprosy skin lesions. Considering that one of the best characterized functions of piRNAs in humans is posttranscriptional mRNA silencing, their functions are similar to what we have described for miRNAs, including acting on apoptosis, *M. leprae* recognition and engulfment, Schwann cell (SC) demyelination, epithelial–mesenchymal transition (EMT), loss of sensation and neuropathic pain. In addition to new findings on leprosy physiopathology, the discovery of relevant piRNAs involved in disease processes in human skin may provide new clues for therapeutic targets, specifically to control nerve damage, a prominent feature of leprosy that has no currently available pharmaceutical treatment.

## Introduction

*Mycobacterium leprae*, the causative agent of leprosy, is the only known bacterium that infects Schwann cells (SCs) of peripheral nerves. In addition to SCs, *M. leprae* is an obligate intracellular pathogen that infects macrophages and dendritic cells; it mostly affects the skin, mucosa and nerves but may be transported within these cells anywhere in the body^1^. Over 200,000 people have been diagnosed with leprosy every year for the last 10 years, and approximately half of these people are clinically recognized to have some physical disability that may result in prejudice and stigma^2^. It is likely that the number of cases is much higher than officially described, and it is thought that there may be up to 4 million hidden cases of leprosy today waiting to be diagnosed, mainly due to loss of medical expertise and lack of access to the health system^3,4^.

Several piRNomes have been described, and most of these have been related to different forms of cancer. piRNAs are important for gametogenesis, embryogenesis and stem cell maintenance^5^. Therefore, piRNAs may have high importance for clinical implications for a variety of diseases.

Posttranscriptional gene silencing by piRNA is mediated by two mechanisms as follows: (i) piRNAs target transposon sequences found in the 3-UTRs or 5-UTRs of mRNAs; and (ii) if the piRNA is derived from a pseudogene and antisense transcript transcribed from the opposite strand of the endogenous genes, then the piRNA targets the mRNA of the corresponding endogenous gene^5^. The piRDisease database 2019^6^ cites 28 diseases with some proven piRNA interference, but none of them are caused by infectious agents. However, there have been reports of piRNA acting on viral diseases^7,8^. In addition, mapping piRNAs onto specific diseases may increase the availability of possible biomarkers, therapeutic targets^9^ and chemoresistance evaluation^10^, which are critical issues for modern leprosy control^11–13^.

## Results

After quality control, alignment, and transcript quantitation, several small noncoding RNAs (sncRNAs) and other transcript fragments were identified. From these sequences, an average of 26,000 reads per sample were recognized as piRNAs, identifying a total of 337 differentially expressed (DE) piRNAs in leprosy patients. In total, 15, 86 and 69 piRNAs were exclusively expressed in skin lesions from tuberculoid (TT) tissue, lepromatous (LL) tissue and healthy subject (HS, control) tissue, respectively. In addition, 139 piRNAs were expressed in all tissue samples irrespective of whether from healthy or leprosy patient skin tissue, and 28 piRNAs were significantly expressed in more than one group (Supplementary Fig. 1).

**Figure 1:**
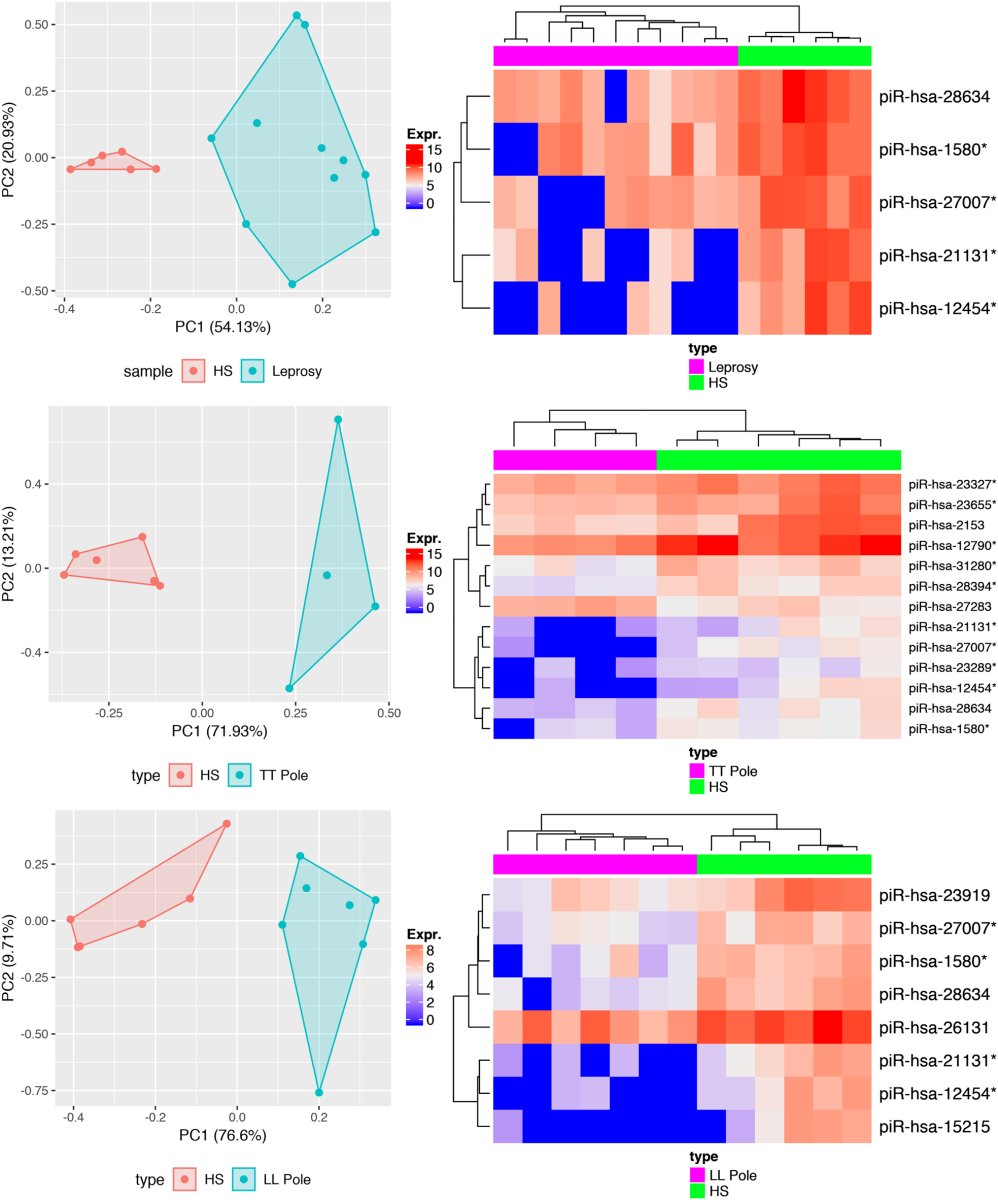
PCAs and heatmaps of differentially expressed piRNAs (|Log2(Fold-change)|> 2; p-value < 0.05) based in RPKM values with the best sensitivity/specificity relation (AUC > 0.9 in ROC analysis). (**A**) Leprosy vs. HS; (**B**) TT Pole vs. HS; and (**C**) LL Pole vs. HS. (*) Indicates colocalized piRNAs.

Sample comparisons were followed by the characterization of the involved piRNAs. Comparison of leprosy patient (TT + LL samples) to HS showed that 14 piRNAs were downregulated in leprosy skin (Supplementary Table 1).From these 14 piRNAs, 5 showed the best sensitivity/specificity relationship in ROC analysis (AUC > 0.9—Fig. 1A, Supplementary Fig. 2), and all 5 of these piRNAs were also DE in other analyses (TT vs. HS and LL vs. HS). In the comparison of TT samples to HS samples, 21 piRNAs were DE. Of these, 20 piRNAs were downregulated in TT, but piR-hsa-27283 was upregulated in TT. From the DE piRNAs, 13 showed the best sensitivity/specificity relationship in ROC analysis (AUC > 0.9—Fig. 1B, Supplementary Fig. 3). In the comparison of LL samples to HS samples, 16 piRNAs were downregulated in LL. Of these 16 DE piRNAs, 8 showed the best sensitivity/specificity relationship in ROC analysis (AUC > 0.9—Fig. 1C, Supplementary Fig. 4). To avoid ambiguous or multimapping identification, colocalized piRNAs were merged (Supplementary Fig. 5).

Aiming to better understand the regulatory role of these 25 downregulated piRNAs (DE with AUC > 0.9), we examined potential piRNA targets using the miRanda^14^ program. Because gene silencing with piRNAs may occur through several mechanisms^5^, we used both 3′ and 5′ UTR regions of genes in miRanda (Supplementary Fig. 6).

**Figure 2:**
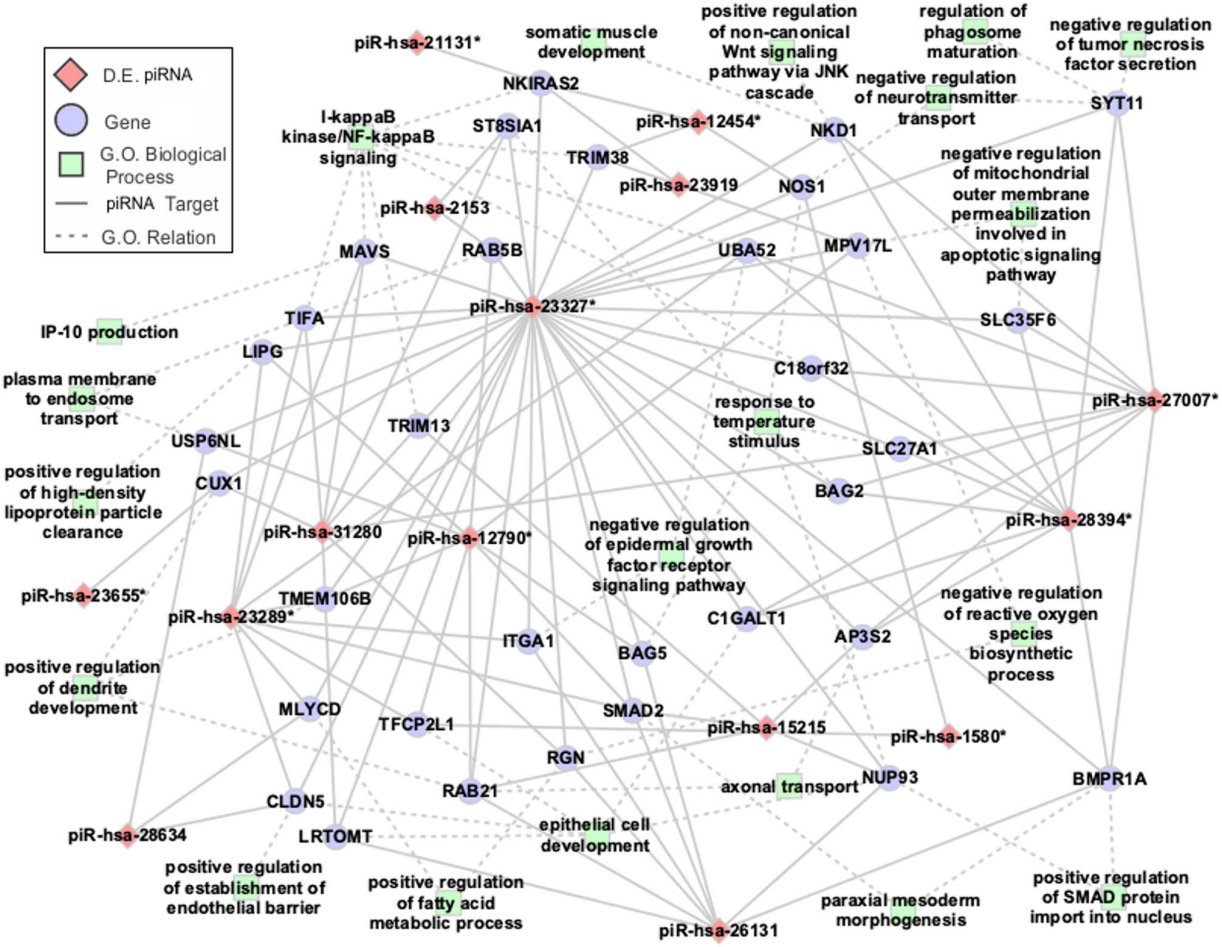
Red indicates differentially expressed piRNAs, and blue indicates potential simultaneous mRNA targets. Green indicates biological processes significantly associated with leprosy phenotype (p-value < 0.05 in gene ontology enrichment). mRNAs were selected based on the following two criteria: (i) target of at least three DE piRNAs and (ii) involved in at least one biological process significantly associated with leprosy phenotype. (*) Indicates colocalized piRNAs.

We identified over 5,000 putative target genes, and 955 of these target genes were targeted simultaneously in both their 5′ and 3′ regions. Using gene ontology (GO; Supplementary Fig. 7), we selected 174 genes that were the putative targets of at least three piRNAs simultaneously (Supplementary Table 2, Supplementary Table 3), and we performed GO enrichment aiming to identify biological processes significantly (p-value < 0.05) involved in the leprosy phenotype (Fig. 2, Supplementary Fig. 7).

DE piRNAs in skin lesions allowed the identification of piRNAs that differentiate LP from HS and also differentiate LP poles, TT and LL from HS. The DE piRNAs were all downregulated in all comparisons (except piR-hsa-27283 in TT vs. HS). In silico analyses of targets and GO enrichment (considering only the posttranscriptional function of piRNAs) revealed genes and pathways important in the leprosy phenotype and the mechanism of immunophysiopathology.

## Discussion

A recent finding of an unexpected role for piRNA in somatic cells has revealed that the capacity to promote sensory axon regeneration after injury may be blocked by piRNA. The piRNA pathway acts against axon regrowth independent of its return to the nucleus and transcriptional silencing. However, with posttranscriptional gene silencing, piRNA inhibition of axonal regeneration is blocked, and sensory nerve regeneration occurs^15^.

**Figure 3:**
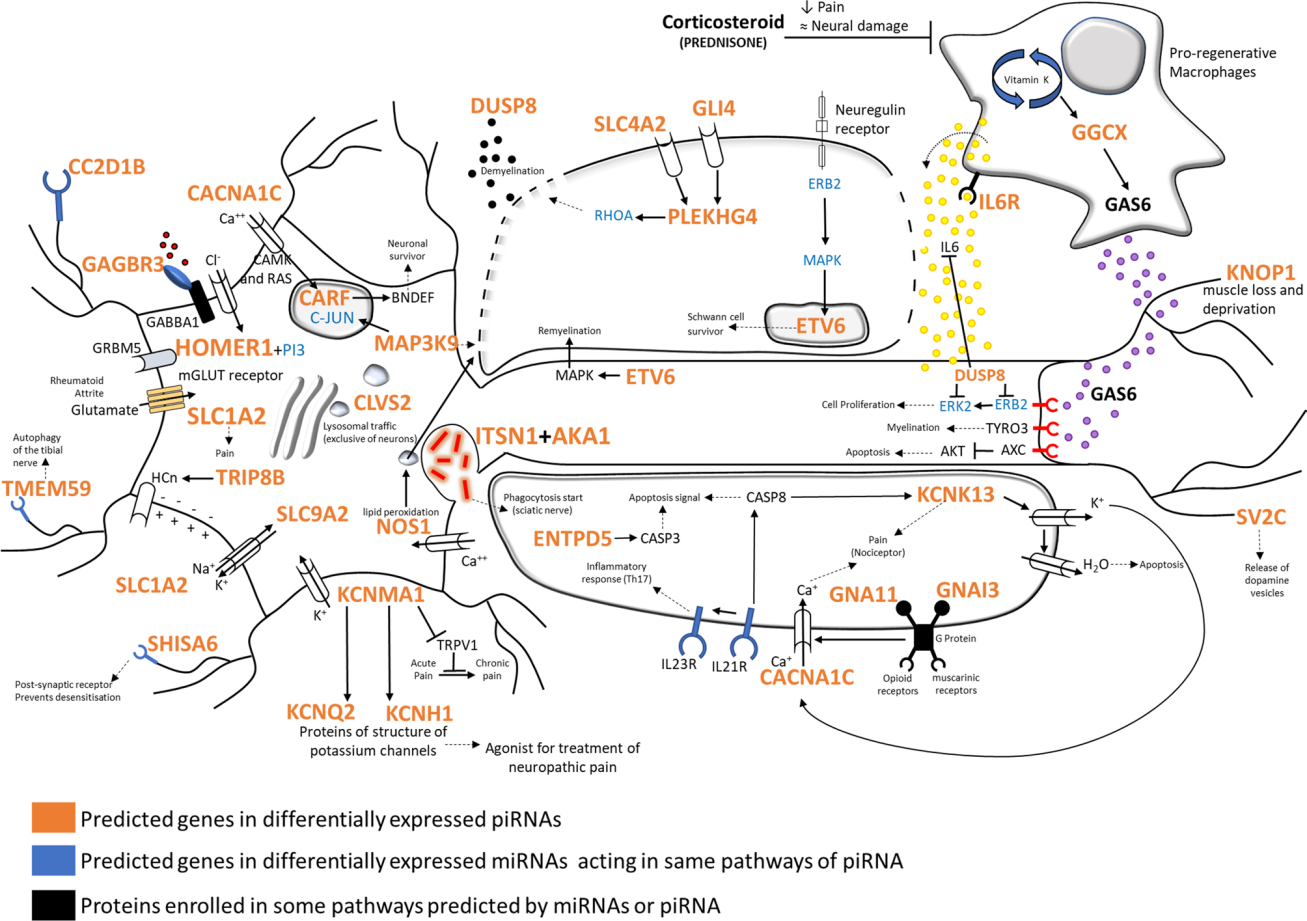
piRNAs related to the epigenetic control of pathways involved in vesicles transport, phagocytosis, demyelination, apoptosis, inflammation, loss of sensation, and pain are differentially expressed in leprosy patient (LP). DE piRNAs do not act on the same targets of miRNA, but they synergistically regulate the same pathway, e.g., infection route by AKA1, TMEM59 and ITSN1 involved in mycobacterial endocytosis in neurons and anti-apoptotic pathways (CARF). Skin anesthesia or hypoesthesia and pain are characteristic of leprosy and have been related to calcium and potassium channel proteins, including SHISA6, KCNMA1, KCNQ2, KCNH1, CACNA1C, SLC1A2 and KCNK13, which are all targets of piRNAs DE. Inflammation and neuropathic pain are part of leprosy clinical aspects and may be moderated by MAPK, HOMER1, PLEKHG4, SLC4A2, GLI4, and DUSP8 by RHOA pathways. ETV6 and CARF are associated with neural regeneration and differentiation of stem cells into Schwann cells. Other important mechanisms of regulating SC maturation and axon regeneration after nerve injury are promoted by pro-regenerative macrophages and are dependent on GGCX, vitamin K, IL-6R and DUSP8 induced by GAS6 stimulation. Pro-regenerative macrophages clear debris in the nerve microenvironment and are essential for axon regeneration. The permanence of pro-regenerative macrophages in the lesional microenvironment promote remyelination and SC differentiation, reducing immature SC density.

Our results demonstrated that, with one exception, all DE piRNAs are downregulated, indicating that targeted genes involved in regeneration of peripheral nerves infected by *M. leprae* may be silent or expressed at low levels (Fig. 3). Considering that one piRNA, like the upregulated piR-hsa-27283, may have approximately 3 thousand genes as targets, it is difficult to analyse its functions as a single piRNA. On the other hand, as the only upregulated piRNA on leprosy skin lesions, piR-has-27283 may be useful as a biomarker of disease, a feature that can be tested in the future. Another important mechanism of regulating SC maturation and axon regeneration after nerve injury is promoted by pro-regenerative macrophages that function to clear debris within the nerve microenvironment. These pro-regenerative macrophages are essential for axon regeneration, and their presence in the lesional microenvironment promotes remyelination and SC differentiation, reducing immature SC density^16^. These mechanisms are regulated by two important molecules, namely, growth arrest specific 6 (GAS6) and IL-6, and the pathways of these molecules are regulated by piRNAs as described in (Fig. 3).

GAS6 is involved in the stimulation of cell proliferation and is associated with a variety of diseases^16^. GAS6 can act through Tyro3 (TAM family of receptors) and contribute to cell survival, invasion, migration, chemoresistance, and metastasis, and as a result, several classes of TAM inhibitors are being developed in clinical studies^17^. GAS6 is produced by quiescent fibroblasts, osteoblasts, and macrophages through GGCX with vitamin K as a coenzyme to the completion of GAS6 carboxylation^16^. Thus, the regenerative function of macrophages in SCs infected with *M. leprae* may depend on the expression of vitamin K-dependent genes (such as GGCX) that produce GAS6 within the microenvironment to promote regrowth of damaged nerves^15^.

In the present study, the downregulation of piRNAs target to IL6R can allow the expression of IL6R in the membrane of pro-regenerative macrophages. This receptor is necessary for the effective action of IL-6 that has been described in patients with higher titers^18,19^ and as a marker of neuropathic pain in patients with leprosy^20^. Therefore, IL-6 may be related to bette nerve regeneration in addition to the stimulation of a more efficient cellular response under the auspices of a downregulated piRNA profile.

Corticosteroids are used to treat acute nerve damage in leprosy, but they have moderate efficacy in treating nerve function impairment. It has been shown that corticosteroids do not have a superior effect to placebo in improving nerve function^21^. After a demyelinating injury, oligodendrocyte precursor cells (OPCs) become activated and subsequently proliferate and migrate to the lesion site^22^, but they have limited action related to corticosteroid treatment due to inhibition of pro-regenerative macrophage uptake^23,24^. Pro-regenerative macrophages persist at the site of the injured nerve and regulate SC maturation and conduction velocity postinjury by a complex GAS6^15,16^-dependent transcriptional profile via GGCX and vitamin K pathways. Increased GAS6 has been described in type 2 reactional episodes of erythema nodosum leprosum (ENL)^25^. The downregulated expression of the piRNAs that control GAS6 production in LP may be related to higher levels of GAS6 in ENL.

Three studies published between 1950 to 1953 showed regression of leprosy nodules in patients treated with vitamin K3 derivatives, which was decades prior to the use of multidrug therapy (MDT) to treat leprosy patients^26–28^. These studies evaluated the daily injection of 50 mg of vitamin K into patients experiencing leprosy reaction, and they reported an extraordinarily rapid improvement within five days with a lowering of fever, alleviation of pain symptoms and full recovery of the patient. Within fifteen days of this treatment, all symptoms of the reactional episode had disappeared without any infiltration. After eight months, the skin lesions disappeared completely and did not persist, without any tingling. Later examination of nasal swab and skin biopsies for the presence of acid-fast bacilli were found to be negative. Based on the interaction of GAS6 and vitamin K pathways, these reports of relief from the often debilitating symptoms of ENL and rapid improvement of skin lesions prior to the use of MDT and corticosteroids suggest the possibility that piRNAs may be involved in posttranscriptional mechanisms promoting migration of pro-regenerating macrophages to the site of nerve injury followed by regrowth of damaged nerves.

The absence of piRNAs allows peripheral nerve regeneration^29–32^. According to the present data, all DE piRNAs in leprosy lesions are downregulated, suggesting that nerve regeneration is not inhibited by piRNAs in leprosy patients. Furthermore, the present work discloses new mechanisms involved on leprosy physiopathology and reveals novel therapeutic targets involved in neurodegeneration and neuropathic pain.

## Methods

### Biological material and clinical data collection

In total, 17 lesion tissue samples were collected as follows: (i) 6 from healthy subjects (HS); (ii) 6 from lepromatous leprosy patients (LL), and (iii) 5 from tuberculoid leprosy patients (TT). All samples were obtained prior to MDT at URE Dr. Marcello Candia (URE) located in the city of Marituba (Pará, Brazil). Immediately after collection, all samples were frozen and stored in RNAlater (SIGMA R0901). Informed consent was obtained from all individual participants.

### RNA extraction and piRNA library preparation

Total RNA was extracted using TRIzol (Thermo Fisher Scientific, Waltham, MA, USA). After isolation, total RNA was stored at − 80 °C until further analysis. Total RNA amount and integrity were determined using the Qubit R 2.0 (Life Technologies, Foster City, CA, USA) and Agilent 2,200 TapeStation (Agilent Technologies, USA) according to the manufacturer’s specifications. Samples with concentrations above 100 μg/uL and RIN (RNA Integrity Number) > 5 were used for sequencing. The concentration of 1 μg/5 μL of sample was used as input to library preparation. We synthesized 17 libraries using the TruSeq Small RNA Library Preparation Kit (Illumina R, San Diego, CA, USA) according to the manufacturer’s instructions. The pool of libraries was quantified using ABI 7,500 equipment (Applied Biosystem, CA, USA) and a KAPA Library Quantification Kit (KAPA Biosystems, Woburn, MA). The libraries were sequenced using a MiSeq Sequencing System (Illumina R, San Diego, CA, USA) and the MiSeq Reagent Kit v3 150 cycle (Illumina R, San Diego, CA, USA).

### NGS read quality control, alignment and quantitation

After sequencing, resulting reads were preprocessed and quality filtered (QV > 25). STAR Aligner (v. 2.4.0.1) was used to map the reads to the human genome (v. hg19)^33^. Htseq-count software^34^ with piRbase annotation^35^ was used to quantify transcripts. Before piRNA quantitation, we grouped colocalized piRNAs to avoid ambiguous counting. Differential expression analysis. To identify the differentially expressed (DE) piRNAs, the following four analyses were performed: (i) leprosy vs. HS samples, (ii) TT vs. HS samples, (iii) LL vs. SH and (iv) TT vs. LL samples. For these analyses, raw read count (read counts 10 in, at least, one sample) were used with DESeq2 library^34^ in the statistical platform R (R Core Team, 2017). piRNAs satisfying the following criteria were tagged as differentially expressed: |Log2 (fold-change)|> 2 and p-value < 0.05. For graphical analysis of piRNAs, expression data were normalized to RPKM. The area under the curve (AUC > 0.9) from the Receiver Operating Characteristic (ROC) analysis was used to identify potential biomarkers among DE piRNAs. All statistical analyses were performed on the platform R.

### Identification of DE piRNA target RNAs

piRNA mRNA targets were identified by predicting the complementarity sequence between each piRNA and the 5-UTRs or 3-UTRs regions of all known human mRNAs (RefSeq gene annotations, Human genome assembly, hg19) with miRanda [v3.3a], applying a stringent alignment score (sc; 170), energy threshold (free energy-30.0 kcal/mol) and at least 80% of complementarity. Functional analysis was performed using cluster profiler library^36^ in R^37^ to identify all biological processes significantly associated (p 0.05) to piRNA-targeted mRNAs5. PCAs and heatmaps were created in R, and the piRNA-gene interaction network was constructed using Cytoscape software v 3.2.1^38^.

Ethics statement. This study was performed in accordance with the recommendations of the Brazilian National Ethics Committee (CONEP) guidelines approved by Pará Federal University Ethics Committee number CAAE 26765414.0.0000.0018 with written informed consent from all subjects, in accordance with the Declaration of Helsinki. The protocol was approved by the Pará Federal University Ethics Committee.

## Supplementary information

Supplementary information.

## Data Availability

The small RNAseq number register is ERP105473 on European Nucleotide Archive database.
