# Supplementary material for: Leprosy piRnome: exploring new possibilities for an old disease
Source: Sci Rep. 2020 Jul 28;10:12648. doi: 10.1038/s41598-020-69355-7 (PMC7387468; doi:10.1038/s41598-020-69355-7)
Supplement: Supplementary file 1 — Supplementary information. [file 41598_2020_69355_MOESM1_ESM.pdf]

Leprosy piRnome: exploring new possibilities for an old disease

Pablo Pinto[1,2,+], Moises Batista da Silva[3+], Fabiano Cordeiro Moreira[2,+], Raquel Carvalho Bouth[3], Ange´lica Rita Gobbo[3], Tatiana Vinasco Sandoval[1], Andre´ Mauricio Ribeiro-dos-Santos[1], Amanda Ferreira Vidal[1], Josafa Gonçalves Barreto[4], Sidney Santos[1,2], John Stewart Spencer[5], Claudio Guedes Salgado[3,*], and Ândrea Ribeiro-dos-Santos[1,2,*]

[1]Human and Medical Genetics Laboratory, Institute of Biological Sciences (ICB), UFPA, Belém, 66075110, Brazil

[2]Oncology Research Center (NPO), UFPA, Belém, 66075110, Brazil

[3]Dermato-Immunology Laboratory, Institute of Biological Sciences (ICB), UFPA, Marituba, 67200000, Brazil

[4]Laboratory of Space Epidemiology (LabEE), UFPA, Castanhal, 68746000, Brazil

[5]Mycobacteria Research Laboratories, Department of Microbiology, Immunology and Pathology, Colorado State University, Fort Collins, 80523-1601, United States

[*]corresponding authors: claudioguedessalgado@gmail.com, akelyufpa@gmail.com

[+]these authors contributed equally to this work

piRNAs	Lep_vs_HS	LL_vs_HS	TT_vs_HS	auc_LL	auc_TT
piR-hsa-1043*	down	down	down	-	-
piR-hsa-1191*	down	down	down	-	-
piR-hsa-12454*	down	down	down	AUC>0.9	AUC>0.9
piR-hsa-12790*	-	-	down	-	AUC>0.9
piR-hsa-15215	down	down	down	AUC>0.9	-
piR-hsa-1580*	down	down	down	AUC>0.9	AUC>0.9
piR-hsa-20613*	down	down	-	-	-
piR-hsa-21131*	down	down	down	AUC>0.9	AUC>0.9
piR-hsa-2153	-	down	down	-	AUC>0.9
piR-hsa-23289*	down	-	down	-	AUC>0.9
piR-hsa-23327*	-	-	down	-	AUC>0.9
piR-hsa-23330*	-	-	down	-	-
piR-hsa-23655*	-	down	down	-	AUC>0.9
piR-hsa-23919	down	down	down	AUC>0.9	-
piR-hsa-26131	-	down	-	AUC>0.9	-
piR-hsa-27007*	down	down	down	AUC>0.9	AUC>0.9
piR-hsa-27282	down	down	down	-	-
piR-hsa-27283	-	-	up	-	AUC>0.9
piR-hsa-27728	down	down	down	-	-
piR-hsa-28212	down	down	down	-	-
piR-hsa-28394*	-	-	down	-	AUC>0.9
piR-hsa-28634	down	down	down	AUC>0.9	AUC>0.9
piR-hsa-31280*	-	-	down	-	AUC>0.9

Supplementary Table 1: piRNA expression profile. (*) Indicates colocalized piRNAs.

Gene Ontology	Description	GeneRatio	pvalue	geneID	Count
GO:1901029	negative regulation of mitochondrial outer membrane permeabilization involved in apoptotic signaling pathway	2/153	0,00184	MPV17L/SLC35F6	2
GO:0048227	plasma membrane to endosome transport	2/153	0,00184	RAB5B/USP6NL	2
GO:0048340	paraxial mesoderm morphogenesis	2/153	0,00236	SMAD2/BMPR1A	2
GO:0060391	positive regulation of SMAD protein signal transduction	2/153	0,00425	BMPR1A/NUP93	2
GO:0051589	negative regulation of neurotransmitter transport	2/153	0,01535	NOS1/SYT11	2
GO:0071612	IP-10 production	1/153	0,00827	MAVS	1
GO:1901231	positive regulation of non-canonical Wnt signaling pathway via JNK cascade	1/153	0,00827	NKD1	1
GO:1905162	regulation of phagosome maturation	1/153	0,00827	SYT11	1
GO:0007249	I-kappaB kinase/NF-kappaB signaling	7/153	0,00573	NKIRAS2/UBA52/TRIM38/TIFA/MAVS/TRIM13/C18orf32	7
GO:1903427	negative regulation of reactive oxygen species biosynthetic process	2/153	0,03037	MPV17L/RGN	2
GO:0009266	response to temperature stimulus	5/153	0,02951	SLC27A1/NOS1/BAG5/BAG2/ST8SIA1	5
GO:0007525	somatic muscle development	1/153	0,03269	NKD1	1
GO:0010983	positive regulation of high-density lipoprotein particle clearance	1/153	0,04863	LIPG	1
GO:1900006	positive regulation of dendrite development	3/153	0,01955	RAB21/TMEM106B/CUX1	3
GO:1904468	negative regulation of tumor necrosis factor secretion	1/153	0,06431	SYT11	1
GO:0045923	positive regulation of fatty acid metabolic process	2/153	0,03386	MLYCD/RGN	2
GO:0002064	epithelial cell development	5/153	0,02646	TFCP2L1/LRTOMT/C1GALT1/NUP93/CLDN5	5
GO:1903142	positive regulation of establishment of endothelial barrier	1/153	0,04069	CLDN5	1
GO:0098930	axonal transport	2/153	0,06894	RAB21/AP3S2	2
GO:0042059	negative regulation of epidermal growth factor receptor signaling pathway	1/153	0,29483	ITGA1	1

Supplementary Table 2: Biological processes associated with leprosy phenotype overrepresented in Gene Ontology enrichment analysis.

piRNAs	Genes
piR-hsa-1043	PIK3IP1, GLCCI1, PDE12, SLC1A2, CREB5, IDS, SLC35F6, SRGAP1, KIAA1958, GNAI3, CC2D1B, NCKAP1, SGPL1, FOXRED2, DIPK2B, TMEM106B, ZNF557, HEBP2, SLC9A7, RPS6KA5, PLEKHG4, MYLK
piR-hsa-1191*	FOXRED2, HELB, SMAD2, MLYCD, TFCP2L1, ZNF276, ZNF618, SRGAP1, SUPT7L, CRKL, VSTM4, GNAI3, NCKAP1, SGPL1, KLRD1, SLC35B4, LRRC3B, MAVS, TFDP2, FRK, SLC9A7, RNF187
piR-hsa-12454*	ZNF276, NOS1, NKIRAS2, SLC1A2, FANCF, PUS7L, TRIM38, NCKAP1, TOGARAM2, ZNF200, KCNQ2, KCNMA1, SLC9A7, SUPT7L
piR-hsa-12790*	PIK3IP1, GGCX, CAPN7, HELB, DTWD1, SOGA1, RAB21, LIPG, CLVS2, SMAD2, SRD5A1, TFCP2L1, KCNH1, ZNF562, PDP2, BAG5, HSPA4L, ZNF385D, MPV17L, FANCF, PUS7L, IL6R, KIAA1958, ARPIN, GNAI3, PLEKHG4, RAB5B, ITSN1, ETV6, USP8, TMEM106B, METTL15, ZNF557, ATXN7L3B, MAP3K9, TMED3, MRPL42, MRPS21, ZNF780B, USP6NL, GABRG3, RPS6KA5, CARF
piR-hsa-15215	KNOP1, RAB21, CLVS2, SMAD2, ZNF276, ZNF83, NUP93, CARF, AP3S2, DUSP8, TRIM13, TFDP2, PLEKHG4, MYLK
piR-hsa-1580*	SRD5A1, TFCP2L1, RNF213, NOS1, OGFOD3, DMAC2L, CREB5, RYBP, ZNF780B, DTWD1
piR-hsa-20613*	FKTN, SLC9A2, SOGA1, RAB21, NKD1, SMAD2, ZNF106, ARCN1, ADD2, TFCP2L1, SPAG9, ZNF562, KCNK13, NOS1, PDE12, ARHGAP1, HSPA4L, SLC1A2, ZNF385D, MRAS, CREB5, SRGAP1, FOXK1, GNAI3, SLC44A1, NCKAP1, IL6R, TOGARAM2, SLC35E3, ITSN1, USP8, ST8SIA1, MAVS, TSPAN14, ZNF557, HEMK1, WDR73, TMED3, TFDP2, SV2C, PEX5L, FRK, RPS6KA5, KIAA0513, MRPL42, MRPS21, LPP, GABRG3, SLC9A7, MYLK
piR-hsa-21131*	SOGA1, NKIRAS2, CC2D1B, NCKAP1
piR-hsa-2153	FKTN, H3F3B, HELB, TMEM59, PARP16, ADD2, CREB5, POU2F1, CACUL1, RAB5B, ST8SIA1, MRPL42
piR-hsa-23289*	GNA11, PIK3IP1, KIAA0513, PAIP2B, LIPG, SMAD2, MLYCD, TFCP2L1, WDFY2, KCNK13, FAM241A, ITGA1, PCDH1, RNF187, IL6R, LYRM4, ABL2, ENTPD5, FOXK1, ARPIN, FIBCD1, TIFA, SGPL1, TOGARAM2, DUSP8, SLC35E3, SLC35B4, ITSN1, TMEM106B, RGP1, CACNA1C, MAVS, SHISA6, TSPAN14, HEMK1, WDR73, PEX5L, UBA52, PCYT2, RNF213, LPP, KCNQ2, CHST9, SLC9A7, CLVS2, TRIM66, SLFNL1, CERCAM, SLC1A2, CLDN5, MAVS, MYLK, SLC35C1
piR-hsa-23327*	GNA11, KNOP1, GGCX, SLC9A2, WDFY1, KIAA0513, PAIP2B, HELB, DTWD1, TTC21B, SLC27A1, SOGA1, TMEM59, RAB21, LIPG, NKD1, SMAD2, ZNF106, ARCN1, MLYCD, RALGAPB, ADD2, SRD5A1, SPAG9, KCNH1, ZNF562, C8orf37, PDP2, WDFY2, PAK2, ZNF276, RNPS1, TRIM66, NOS1, ADGRE2, PDE12, FAM241A, BAG5, ARHGAP1, NKIRAS2, HSPA4L, LRTOMT, SLC1A2, DTX3L, ZNF385D, OGFOD3, ZNF618, ITGA1, DMAC2L, MPV17L, RNF187, VPS35, FANCF, CUX1, PUS7L, AP3S2, CREB5, MPI, IDS, SLC35F6, SRGAP1, SNX21, SUPT7L, KIAA1958, LYRM4, CRKL, TPGS2, ENTPD5, FOXK1, ARPIN, TRIM38, VSTM4, GNAI3, POU2F1, CC2D1B, SLC44A1, ZNF605, BMPR1A, FIBCD1, CERCAM, SYT11, CACUL1, RAB5B, BAG2, SGPL1, FOXRED2, TOGARAM2, SLC35E3, KLRD1, TRIM13, SLC35B4, ITSN1, DIPK2B, ETV6, USP8, ST8SIA1, TMEM106B, RGP1, CACNA1C, MAVS, C1GALT1, TSPAN14, ZNF557, HEMK1, CCDC141, WDR73, TMED3, ATXN7L3B, RYBP, ABL2, TFDP2, RPP14, FRAS1, SV2C, MFN1, UBA52, PCYT2, HEBP2, NUP93, MRPL42, RNF213, LPP, KCNQ2, KCNMA1, ZNF780B, CHST9, USP6NL, GABRG3, C18orf32, SLC9A7, RPS6KA5, ZBED4, HEMK1, GLCCI1, CAPN7, PARP16, KCNK13, DNAJB5, PCDH1, PROCA1, CC2D1B, SLC35C1, BAG5, ATP5MC3, PCYT2, IL6R, SLFNL1, RGN, HOMER1, TIFA, CERCAM, TMEM59, PLEKHG4, TRIM13, SLC35B4, KLRD1, TSPAN14, MRAS, ZNF200, LRRC3B, MYLK, CLDN5, PIK3IP1, ZNF83, KIAA0513, MAP3K9, USP6NL
piR-hsa-23330*	FOXK1, CHST9
piR-hsa-23655*	FRAS1, KCNK13, PDE12, HSPA4L, ZNF385D, CUX1, VSTM4, SV2C, FRK, HEBP2, CHST9
piR-hsa-23919	SOGA1, TMEM59, C8orf37, RPP14, PDE12, FAM241A, NKIRAS2, OGFOD3, MPV17L, IDS, SRGAP1, KIAA1958, TRIM38, NCKAP1, CACUL1, SLC35E3, SLC35B4, USP8, METTL15, ZNF557, WDR73, RYBP, TFDP2, FRK, HEBP2, RNPS1, CERCAM, ZNF83
piR-hsa-26131	KNOP1, CAPN7, KIAA0513, PAIP2B, HELB, TMEM59, RAB21, CLVS2, SMAD2, C8orf37, TRIM66, LRTOMT, BAG5, ITGA1, DMAC2L, PUS7L, IDS, FOXK1, VSTM4, GNAI3, CACNA1C, SLC35E3, KLRD1, PCDH1, RGP1, METTL15, HEMK1, TMED3, ATXN7L3B, SV2C, NUP93, MRPL42, LPP, GABRG3, RNF187, ATP5MC3, BMPR1A, RGN
piR-hsa-27007*	OTUB2, ZBED4, GGCX, FKTN, SLC9A2, WDFY1, H3F3B, TTC21B, SLC27A1, NKD1, ZNF106, ARCN1, RALGAPB, MPI, PAK2, PROCA1, RNPS1, DNAJB5, ADGRE2, ARHGAP1, DTX3L, ZNF618, VPS35, MRAS, AP3S2, KLRD1, SLC35F6, SNX21, HOMER1, CRKL, ABL2, TPGS2, CC2D1B, SLC44A1, ZNF605, BMPR1A, SYT11, BAG2, FOXRED2, DUSP8, DIPK2B, LRRC3B, SHISA6, C1GALT1, CCDC141, FRAS1, LYRM4, MFN1, PEX5L, UBA52, MRPS21, KCNMA1, C18orf32, RPS6KA5, GLCCI1, PDP2, SPAG9
piR-hsa-27282	CLVS2, TIFA, FRK
piR-hsa-27283	FOXRED2, SMAD2, PUS7L, LYRM4, POU2F1, NCKAP1, BAG2, TRIM13, HEMK1, RNF213, KCNMA1, USP6NL
piR-hsa-27728	SRD5A1, ARPIN
piR-hsa-28212	SRGAP1
piR-hsa-28394*	OTUB2, ZBED4, GGCX, FKTN, SLC9A2, WDFY1, H3F3B, TTC21B, SLC27A1, NKD1, ZNF106, ARCN1, RALGAPB, MPI, PAK2, PROCA1, RNPS1, DNAJB5, ADGRE2, ARHGAP1, DTX3L, ZNF618, VPS35, MRAS, AP3S2, KLRD1, SLC35F6, SNX21, HOMER1, CRKL, ABL2, TPGS2, CC2D1B, SLC44A1, ZNF605, BMPR1A, SYT11, BAG2, FOXRED2, DUSP8, DIPK2B, LRRC3B, SHISA6, C1GALT1, CCDC141, FRAS1, LYRM4, MFN1, PEX5L, UBA52, MRPS21, KCNMA1, C18orf32, RPS6KA5, GLCCI1, PDP2, SPAG9
piR-hsa-28634	GNA11, MLYCD, SLFNL1, CLDN5, PCYT2, FRK, RNF213, USP6NL, OTUB2, ADD2, PLEKHG4, ZNF200, SLC35C1, RPP14
piR-hsa-31280	HEMK1, KIAA0513, SLC27A1, PARP16, KCNH1, ZNF562, ATP5MC3, WDFY2, LRTOMT, CARF, CUX1, MPI, SRGAP1, SUPT7L, RGN, POU2F1, FIBCD1, TIFA, SGPL1, SLC35E3, ZNF200, TRIM13, ETV6, ST8SIA1, MAVS, TSPAN14, ENTPD5, MAP3K9, RPS6KA5, CERCAM

Supplementary Table 3: Potential targets of at least three DE piRNAs.

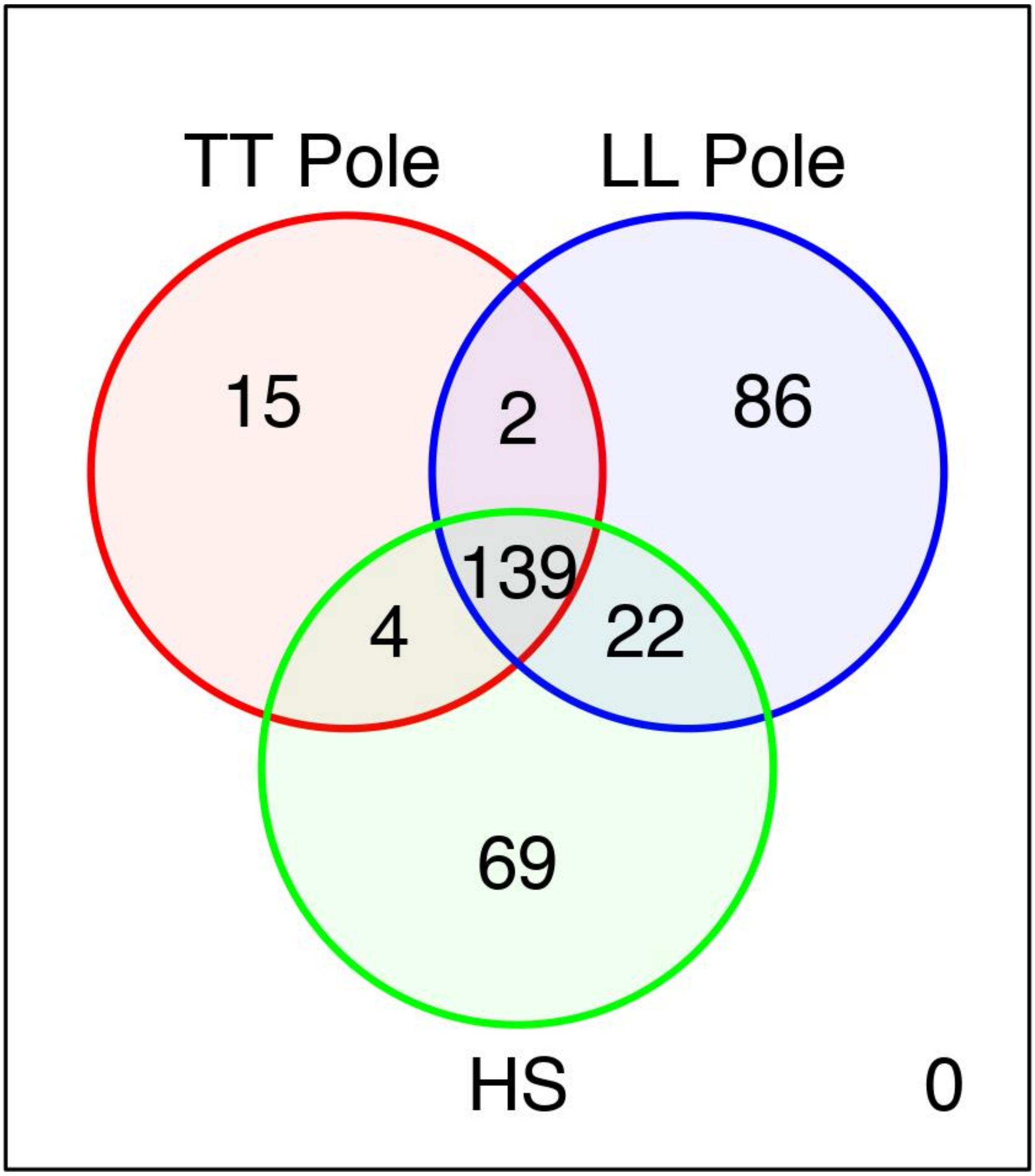

Supplementary Figure 1: Venn Diagram with piRNAs distribution among tissues.

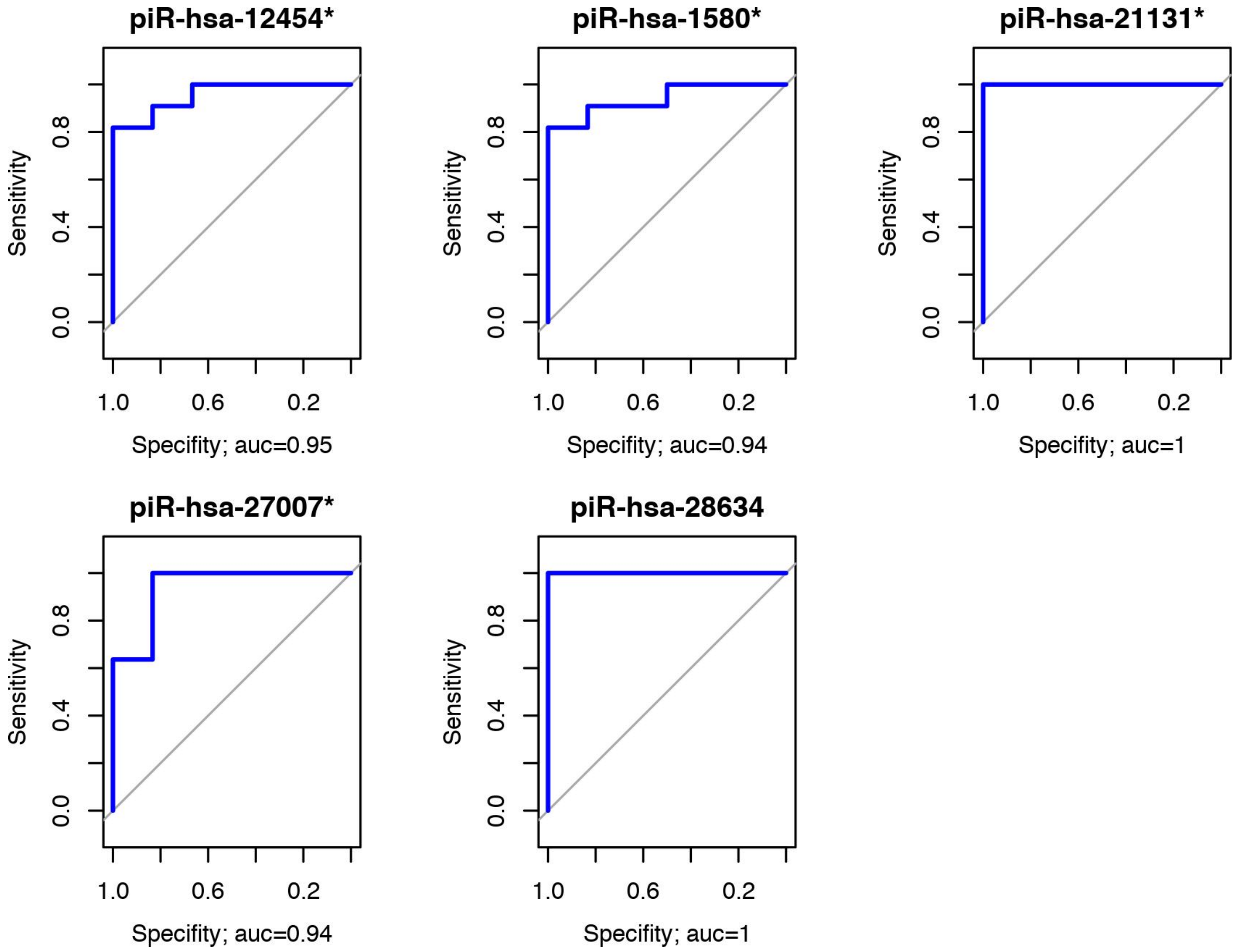

Supplementary Figure 2: piRNAs with the best sensitivity/specificity relation in leprosy vs. HS comparison (ROC analysis; AUC > 0.9). (*) Indicates colocalized piRNAs.

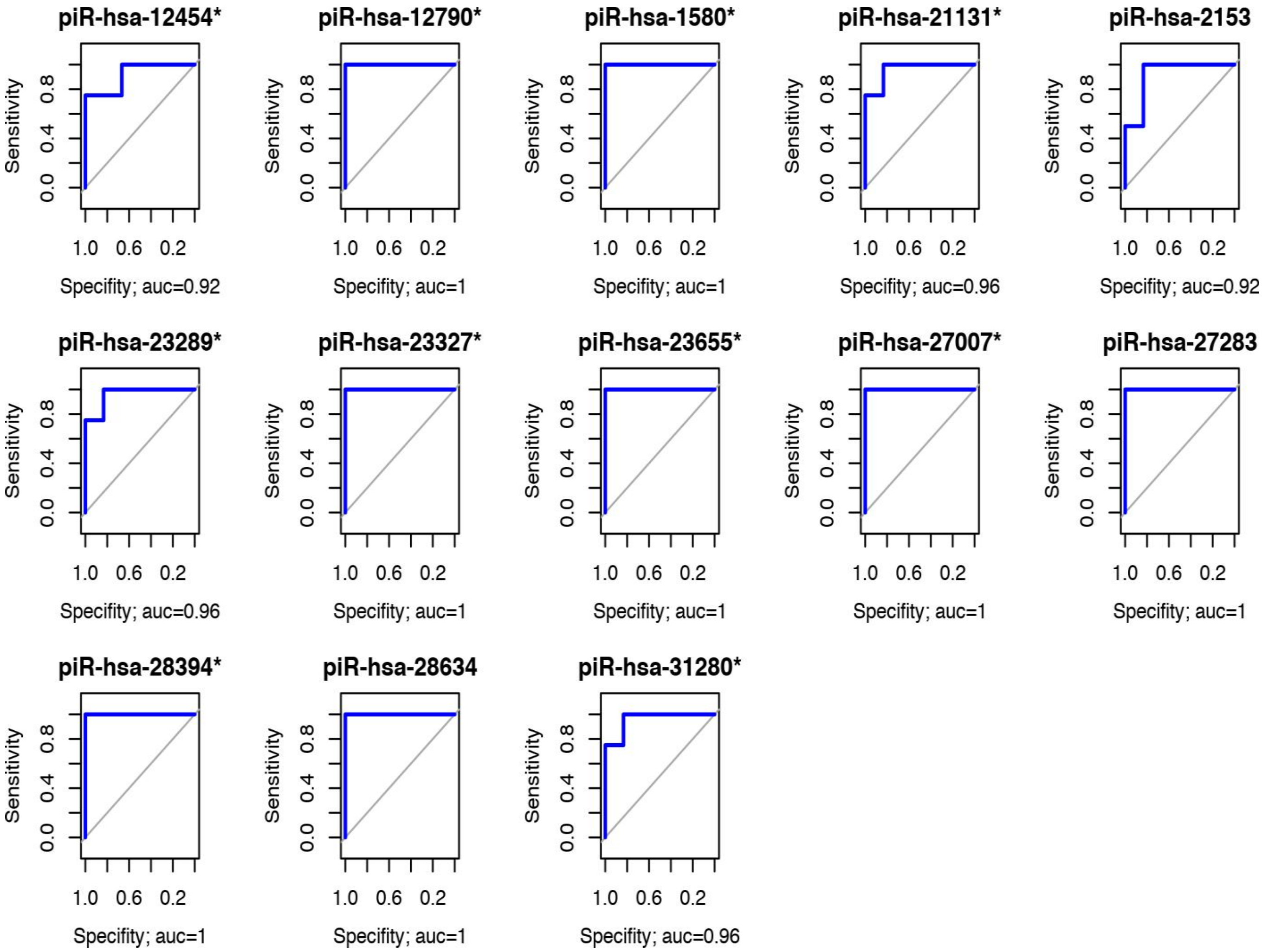

Supplementary Figure 3: piRNAs with the best sensitivity/specificity relation in LL Pole vs. HS comparison (ROC analysis; AUC > 0.9). (*) Indicates colocalized piRNAs.

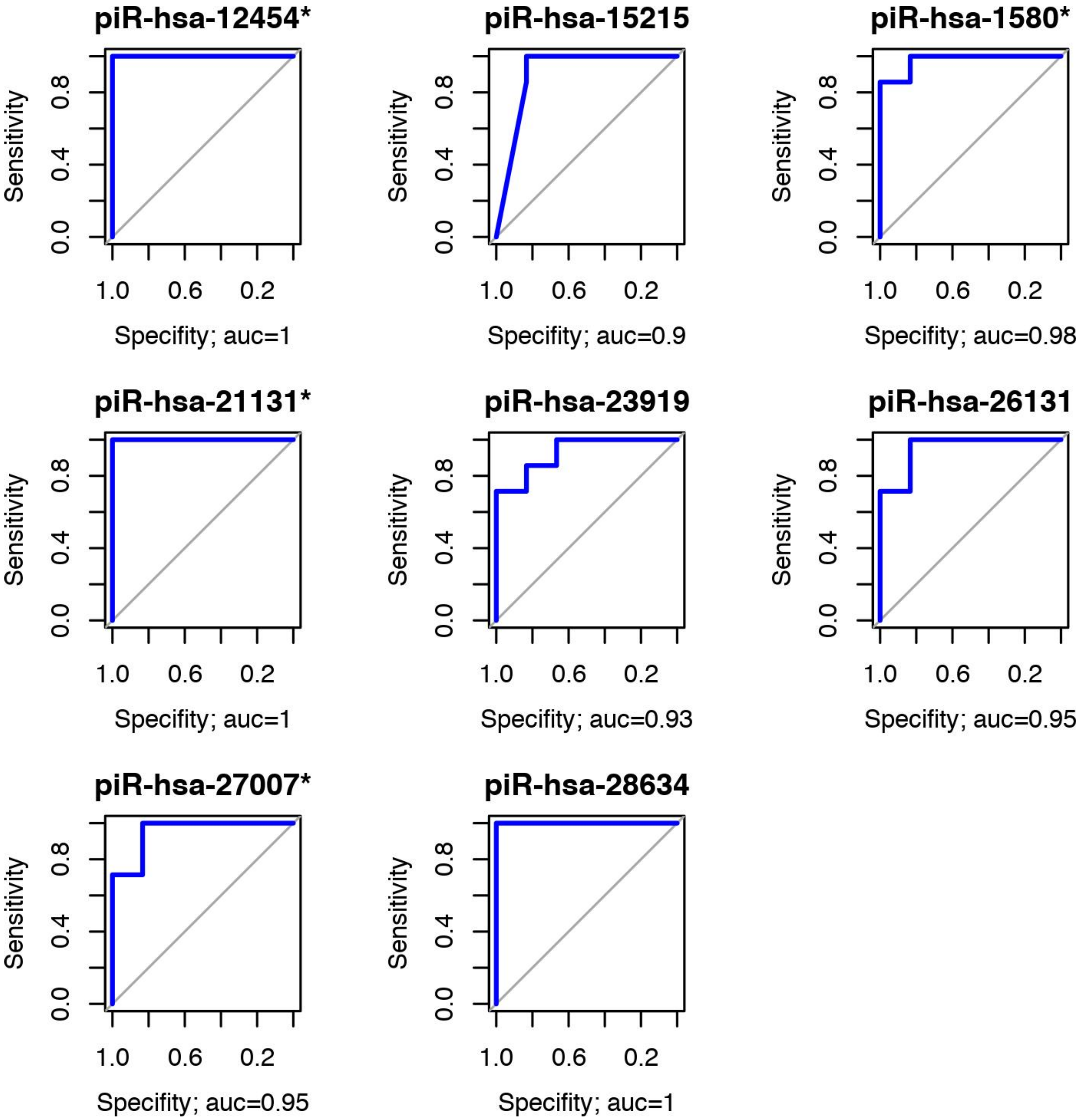

Supplementary Figure 4: piRNAs with the best sensitivity/specificity relation in TT Pole vs. HS comparison (ROC analysis; AUC > 0.9). (*) Indicates colocalized piRNAs.

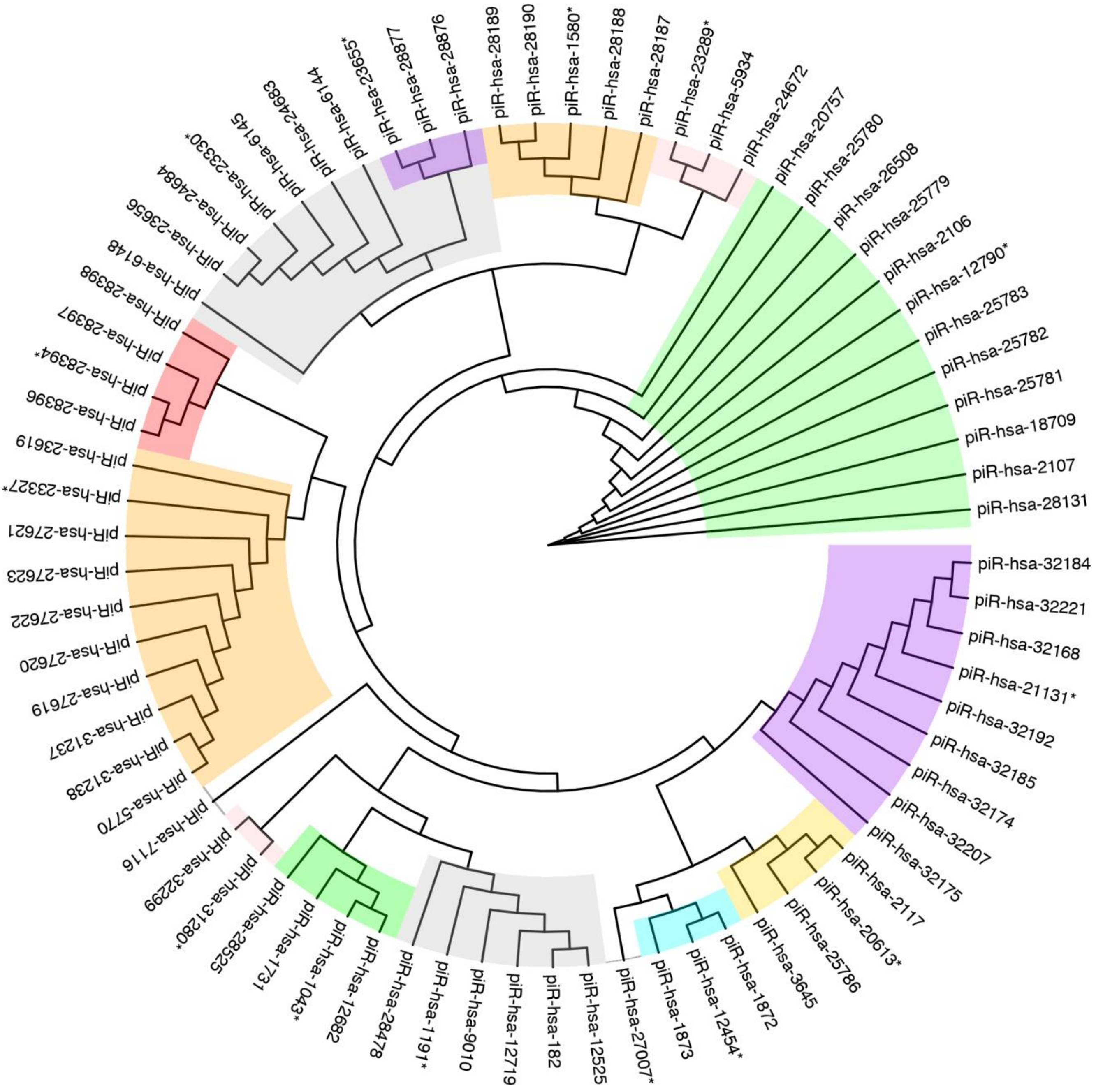

Supplementary Figure 5: Similarity tree based on the nucleotide alignment of colocalized piRNAs merged into 14 groups. (*) Indicates piRNA names used in this work, and the colors indicate all colocalized piRNAs.

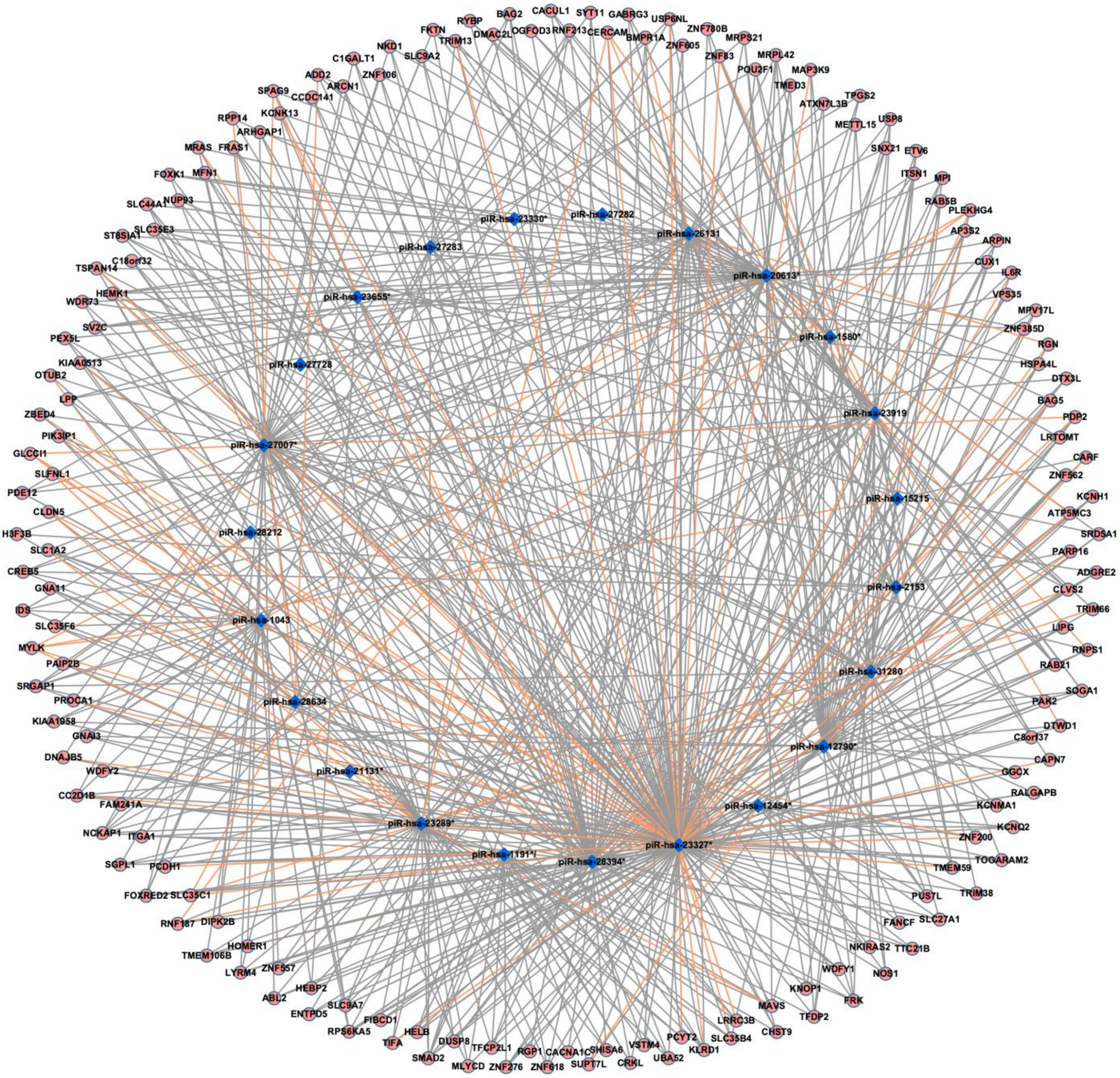

Supplementary Figure 6: Biological processes associated with leprosy phenotype over represented in Gene Ontology enrichment analysis.

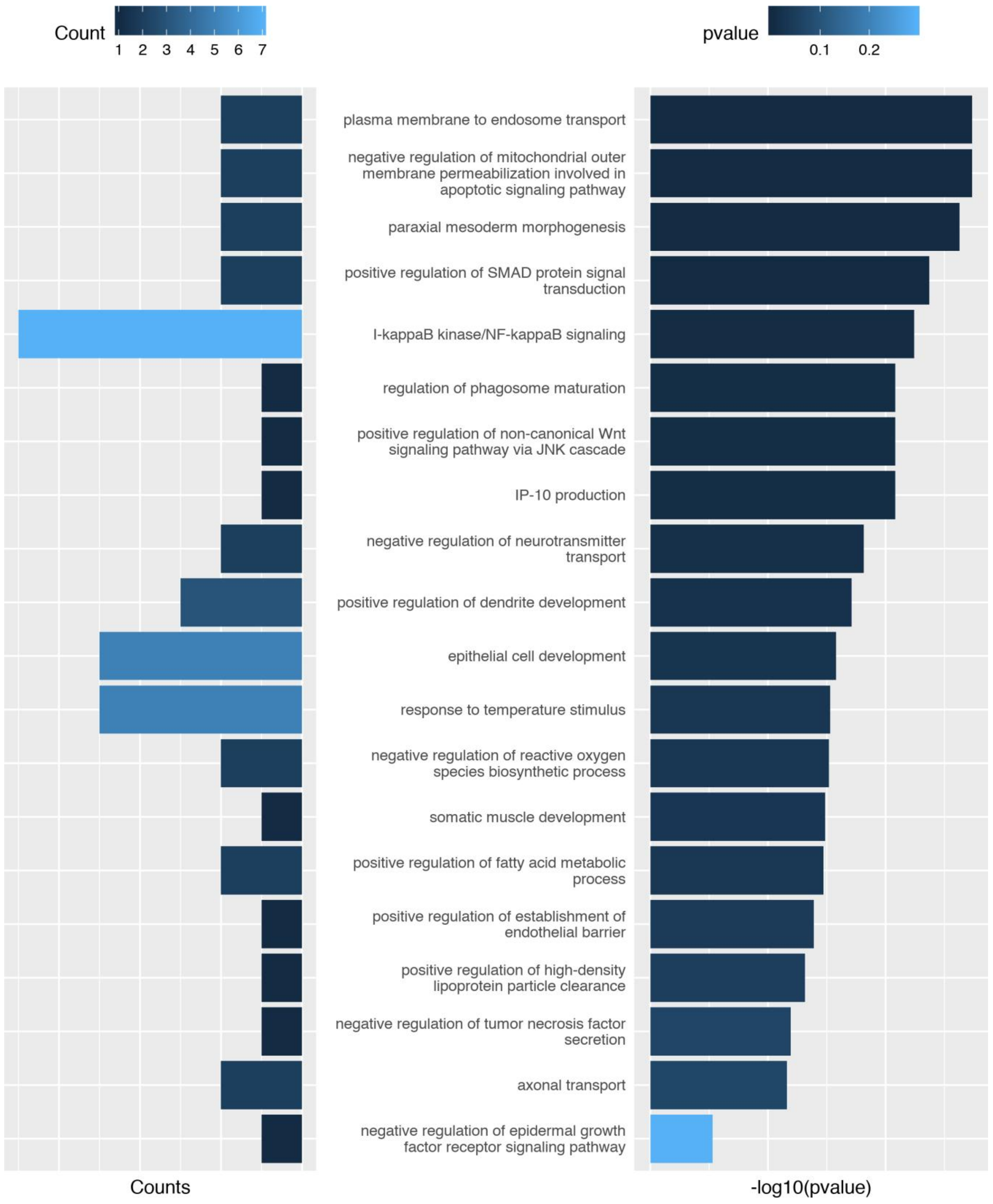

Supplementary Figure 7: Differentially expressed piRNAs and their potential targets. The network shows mRNA targets of at least three piRNAs. Orange lines designate potential 5'UTR regulation, and gray lines designate potential 3'UTR regulation.
